# Combination of Serum Neurofilament Light Chain and Serum Cardiac Troponin T as Biomarkers Improves Diagnostic Accuracy in Amyotrophic Lateral Sclerosis

**DOI:** 10.1002/ana.78066

**Published:** 2025-10-24

**Authors:** Paula Lindenborn, Rachel Fabian, Torsten Grehl, Huelya Nazlican, Thomas Meyer, Sarah Bernsen, Patrick Weydt

**Affiliations:** ^1^ Department of Neuromuscular Diseases Center for Neurology, University Hospital Bonn Bonn Germany; ^2^ Department of Parkinson, Sleep and Movement Disorders Center for Neurology, University Hospital Bonn Bonn Germany; ^3^ Department of Palliative Medicine University Hospital Bonn Bonn Germany; ^4^ Department of Neurology Center for ALS and other Motor Neuron Disorders, Alfried Krupp Hospital Essen Germany; ^5^ Department of Neurology Center for ALS and Other Motor Neuron Disorders, Charité ‐ Universitätsmedizin Berlin, Corporate Member of Freie Universität Berlin, Humboldt‐Universität zu Berlin and Berlin Institute of Health Berlin Germany; ^6^ Ambulanzpartner Soziotechnologie APST Berlin Germany; ^7^ German Center for Neurodegenerative Diseases Bonn Germany

## Abstract

**Objective:**

We aimed to evaluate the clinical utility of serum neurofilament light chain (sNfL) and cardiac troponin T (cTnT) in amyotrophic lateral sclerosis (ALS) and assess whether their combination improves diagnostic accuracy.

**Methods:**

We retrospectively analyzed 293 ALS patients, 85 neurodegenerative disease controls, and 29 healthy controls. A validation cohort of 501 ALS patients was analyzed to confirm reproducibility of the results. Receiver operating characteristic (ROC) curve analysis was performed for sNfL, cTnT, and their combination, and the area under the curve (AUC) was compared across groups. An ALS‐specific cTnT cut‐off of 8.35ng/L was determined using the Youden index and applied in subgroup analyses, in which “biomarker‐negative” ALS patients were compared to “biomarker‐positive” patients regarding disease duration and progression.

**Results:**

sNfL showed excellent performance in discriminating ALS patients from healthy controls (AUC = 0.94), but only moderate performance in discriminating neurodegenerative disease controls (AUC = 0.82). Combining sNfL and cTnT improved diagnostic accuracy for ALS over neurodegenerative controls, with an AUC of 0.90, whereas cTnT alone showed an AUC of 0.77. The validation cohort showed similar AUCs. “Biomarker‐negative” ALS patients had a longer disease duration (73.0 vs 18.0 months, *p* = 0.0003) and a lower progression rate (0.19 vs 0.70 points per months, *p* < 0.0001) than “biomarker‐positive” patients.

**Interpretation:**

Although sNfL alone performs well in distinguishing ALS from healthy controls, repurposing cTnT for ALS provides additional value in discriminating ALS from disease controls. The combination of sNfL and cTnT improves diagnostic accuracy and may help identify prognostically distinct ALS subgroups. ANN NEUROL 2026;99:408–417

Amyotrophic lateral sclerosis (ALS) is a progressive and ultimately fatal neurodegenerative disease characterized by the selective degeneration of upper motor neurons (UMN) and lower motor neurons (LMN).^1^, ^2^ As the disease progresses, symptoms spread and cause generalized weakness, respiratory impairment, and ultimately death because of respiratory failure.^1^ The complex pathophysiology results in both neuroaxonal damage and neuromuscular changes.^3^

Neurofilament light chain (NfL) is a structural protein of the neuronal cytoskeleton that is released into the cerebrospinal fluid (CSF) and blood during axonal stress. Elevated levels of NfL in CSF and serum can be reliably quantified with highly sensitive assays and are strongly associated with neurodegeneration making them reliable biomarkers for ALS.^4^, ^5^, ^6^ NfL in CSF has been extensively studied, numerous studies have demonstrated that serum NfL (sNfL) strongly correlates with CSF levels offering comparable diagnostic performance.^7^ Importantly, sNfL measurement is minimally invasive and, therefore, much more practical for longitudinal monitoring. However, sNfL is not specific to ALS, as elevated levels are also observed in other neurological conditions such as multiple sclerosis, Huntington's disease, spinal cord injury, and many more.^8^, ^9^, ^10^ This limits its utility as diagnostic marker, especially when it comes to differentiating ALS from clinically overlapping disorders.

Troponins are essential structural and functional components of skeletal and cardiac muscle. Two subunits, troponins T and I, come in tissue specific isoforms and assays, and their “cardiac specific” isoforms are in wide clinical use as blood biomarkers of acute myocardial damage.^11^, ^12^ In skeletal muscle diseases, cardiac troponin T (cTnT), but not cardiac troponin I, is expressed ectopically resulting in chronically elevated cTnT blood levels that can be detected in with the “cardiac‐specific” assay.^13^ Recent studies have shown that cTnT can serve as a potential biomarker for skeletal muscle involvement in neuromuscular diseases, including ALS.^14^, ^15^

Longitudinal studies show that cTnT levels increase as ALS progresses and correlate with functional decline, respiratory impairment, and overall disease severity.^16^, ^17^ Importantly, sNfL and cTnT are not correlated with one another, suggesting that they reflect distinct aspects of ALS pathology.^15^

Given the complementary roles of sNfL and cTnT, we hypothesized that the combination of these biomarkers has the potential to improve diagnostic accuracy in ALS. This dual‐marker approach could enhance the differentiation of ALS from related conditions and may help define clinical subgroups with specific disease characteristics.

In this study, we evaluated the diagnostic performance of sNfL and cTnT both individually and in combination, by comparing ALS patients with healthy controls and neurodegenerative controls. By combining both biomarkers, we aimed to strengthen diagnostic accuracy and refine the biological understanding of ALS.

## Patients and Methods

### Study Design and Population

We included in this retrospective analysis a discovery cohort of 293 ALS patients and 2 control groups: 85 neurodegenerative disease controls and 29 healthy controls. In addition, an independent validation cohort consisting of 501 ALS patients from the Alfred Krupp Hospital Essen was included to replicate and strengthen the findings of the primary analysis. The ALS discovery cohort included individuals seen between November 2020 and March 2023, while the validation cohort consisted of patients seen between January 2023 and April 2025. All the ALS patients were diagnosed according to established clinical criteria, and key clinical and demographic data were recorded.

The neurodegenerative disease controls group consisted of patients from our outpatient clinic with neurodegenerative disorders other than classic ALS. This group included individuals with non‐ALS motoneuron diseases (MND) like primary lateral sclerosis (PLS) (n = 8), spinal bulbar muscular atrophy (SBMA) (n = 8), spinal muscular atrophy (SMA) (n = 14), hereditary sensory and motor neuropathy type 1 (HSMN1) (n = 2), 2 cases classified as non‐ALS motor neuron disease on the 1 hand and Huntington's disease (HD) (n = 50), plus 1 case of non‐HD chorea on the other hand. HD was selected as a control condition, as it represents a non‐MND neurodegenerative disorder with a consistently elevated and well‐characterized NfL signal.

The healthy controls included presymptomatic HD (n = 13), as well as individuals diagnosed with benign fasciculation crampus‐syndrome (BFCS) (n = 9) and somatoform disorders (n = 7).

For all participants, demographic data (age, sex, body‐mass‐index [BMI]) as well as serum levels of cTnT and NfL were collected. In ALS patients, the functional status was assessed using the revised ALS functional rating scale (ALSFRS‐R), a validated 12‐item questionnaire evaluating bulbar, fine motor, gross motor, and respiratory function (score range: 0–48). Disease progression was quantified by calculating the progression rate (PR), defined as: PR = (48–ALSFRS‐R at time of assessment)/disease duration in months. For the validation cohort, the same clinical parameters were collected, including age, ALSFRS‐R, disease duration, progression rate and serum NfL and cTnT levels.

As both laboratory measurements and clinical assessments were part of routine diagnostic procedures and analyzed retrospectively, no formal ethical approval was required according to our institutional ethics committee (decision no. 324/20).

### Biomarker Measurements

sNfL was measured using a single‐molecule array (Simoa, Quanterix Corporation, Lexington, MA) in the academic laboratory of the University Hospital Ulm, Department of Neurology (for the discovery cohort) or at the Berlin ALS center (for the validation cohort). sNfL serum concentrations were measured in pg/mL and age‐dependent cut‐off values were applied based on previous studies^18^, ^19^, ^20^: <51 years: 22pg/mL, 51–60 years: 34pg/mL, 61–70 years: 45pg/mL, 71–80 years: 57pg/mL, and 80 years: 78pg/mL.

Serum cTnT was analyzed using a highly sensitive electrochemiluminescence immunoassay (ECLIA) in a commercial, clinically fully accredited laboratory (Labor Volkmann, Karlsruhe, Germany). cTnT serum concentrations were measured in ng/L and to determine the optimal diagnostic cut‐off value for cTnT in ALS, the Youden index (J = sensitivity + specificity – 1) was calculated from the receiver operating characteristic (ROC) analysis.

### Statistical Analysis

As none of the variables showed consistent normal distribution (per D'Agostino‐Pearson omnibus test), continuous variables (such as age, BMI, and biomarker concentrations) are uniformly reported as median and interquartile range (IQR). Group comparisons between ALS patients, neurodegenerative disease controls, and healthy controls were conducted using the Kruskal–Wallis test. Differences in disease duration, progression rate, and other clinical parameters regarding to the analysis of the biomarker‐negative and ‐positive ALS patients were analyzed using the Mann–Whitney *U* test.

ROC curves were generated to evaluate the diagnostic performance of sNfL, cTnT, and their combination.^21^, ^22^ ROC analysis was performed for: ALS cohort/validation cohort versus healthy controls, ALS cohort/validation cohort versus neurodegenerative disease controls, and ALS cohort/validation cohort versus all controls combined.

The area under the curve (AUC) was calculated to assess diagnostic accuracy. Statistical significance was defined as *p* < 0.05. To evaluate the combined performance of both biomarkers, a composite score was generated using logistic regression based on sNfL and cTnT levels. The resulting predicted probabilities were used to generate a ROC curve, which was then compared to the individual ROC curves of sNfL and cTnT.

All statistical analyses were performed with GraphPad Prism 10.2.2 (GraphPad Software, San Diego, CA). Data were stored and managed using Microsoft Excel 2019 (Microsoft, Redmond, WA).

## Results

The clinical and demographic characteristics of ALS patients, neurodegenerative disease controls, and healthy controls are summarized in Table 1. The median age of ALS patients was 67.0 years (IQR: 59.0–74.5), which was significantly higher than the neurodegenerative disease controls (59.0 years, IQR: 55.0–67.0) and the healthy controls (53.5 years, IQR: 44.3–58.8, *p* < 0.0001). The median BMI in the ALS group was 24.2 kg/m^2^ (IQR: 22.1–26.8), which was lower than the neurodegenerative disease controls (25.4 kg/m^2^, IQR: 23.0–27.6) and the healthy controls (29.6 kg/m^2^, IQR: 24.9–32.3, *p* = 0.0457).

**TABLE 1:** Summary of Patient Characteristics

	ALS cohort	ND controls	Healthy controls	*p*	Validation cohort
n	293	85	29	NA	501
Sex, F, n (%)	127 (43.3)	40 (47)	14 (48)	NA	203 (40.5)
Age, yr	67.0 (59.0–74.5)	57.0 (46.0–64.0)	52.0 (39.5–57.0)	<0.0001	63.5 (56.0–71.0), n = 490
BMI (kg/m^2^)	24.2 (22.1–26.8), n = 234	25.4 (23.0–27.6), n = 15	29.6 (24.9–32.3), n = 9	0.0457	NA
ALSFRS‐R	35.0 (29.0–40.0) n = 243	NA	NA	NA	35.0 (28.0–41.0), n = 494
Disease duration	18.0 (9.25–32.8) n = 284	NA	NA	NA	7.00 (2.00–25.5) n = 489
Progression rate, points per month	0.67 (0.34–1.22) n = 240	NA	NA	NA	1.33 (0.52–3.33) n = 455
cTnT median value (ng/L)	18.2 (9.00–33.2)	6.5 (4.45–10.05)	4.0 (3.00–7.15)	<0.0001	20.0 (11.0–36.0)
sNfL median value (pg/mL)	93.0 (55.5–149.5)	40.0 (19.5–57.5)	13.0 (9.00–32.0)	<0.0001	51.5 (27.8–96.1)

All values are presented as median (interquartile range). *p* Values refer to comparisons between ALS patients, neurodegenerative disease controls and healthy controls (Kruskal–Wallis test).

ALS = amyotrophic lateral sclerosis; ALSFRS‐R = Revised Amyotrophic Lateral Sclerosis Functional Rating Scale; BMI = body mass index; F = female; cTnT = cardiac troponin T; NA = not applicable; ND controls = neurodegenerative disease controls; sNfL = serum neurofilament light chain; yr = years.

At baseline, ALS patients had a median ALSFRS‐R score of 35.0 (IQR: 29.0–40.0), a median disease duration of 18.0 months (IQR: 9.25–32.8), and a median progression rate of 0.67 points per month (IQR: 0.34–1.22). The median sNfL level in ALS patients was 93.0pg/mL (IQR: 55.5–149.5), compared to 45.0pg/mL (IQR: 25.0–62.0) in the neurodegenerative disease controls and 12.5pg/mL (IQR: 9.00–29.8) in the healthy controls (*p* < 0.0001). Serum cTnT levels were significantly higher in ALS patients (18.2ng/L, IQR: 9.00–33.2) compared to neurodegenerative disease controls (6.1ng/L, IQR: 3.90–11.0) and healthy controls (4.45ng/L, IQR: 3.00–7.58, *p* < 0.0001). To confirm the robustness of our findings, we also analyzed an independent validation cohort (n_v_ = 501). Compared to the discovery cohort, patients in the validation cohort were slightly younger (median age 63.5 years, IQR: 56.0–71.0) and had a shorter disease duration (7.0 months, IQR: 2.0–25.5). The median progression rate was higher in the validation cohort (1.33 points per month, IQR: 0.52–3.33). ALSFRS‐R scores at baseline were similar between cohorts (median 35.0 in both groups). Median serum cTnT levels were comparable between the 2 cohorts (20ng/L, IQR: 11.0–36.0), whereas median sNfL levels were lower in the validation cohort (51.5 ng/mL, IQR: 27.8–96.1) (Table 1).

**FIGURE 1:**
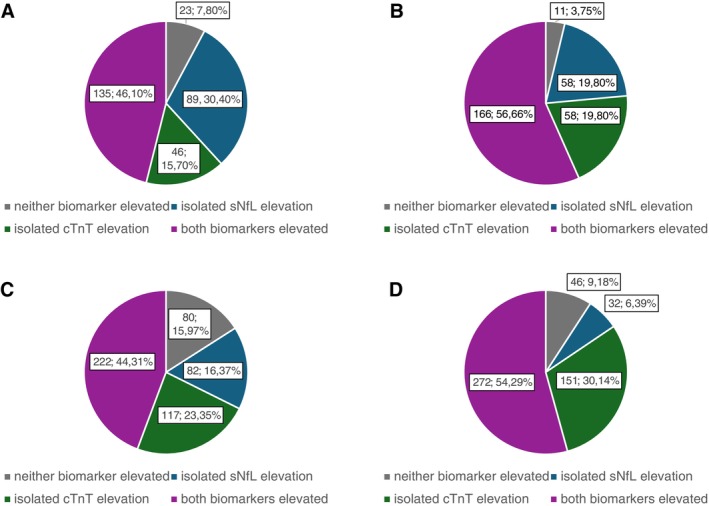
Distribution of biomarker status in amyotrophic lateral sclerosis (ALS) patients of the discovery and the validation cohort using 2 different cardiac troponin T (cTnT) cut‐offs. (A, B) Show classifications in the discovery cohort using the conventional cardiac cut‐off (14ng/L) and the ALS‐specific cut‐off (8.35ng/L). (C, D) Show the corresponding classifications in the validation cohort using the same 2 cut‐offs. In all diagrams, ALS patients are stratified into 4 subgroups based on biomarker status: neither biomarker elevated (normal serum neurofilament light chain [sNfL]) and cTnT; isolated sNfL elevation (elevated sNfL, normal cTnT); isolated cTnT elevation (elevated cTnT, normal sNfL) both biomarkers elevated (sNfL and cTnT elevated); and percentages refer to the proportion of patients in each subgroup relative to the entire ALS discovery cohort (n = 293) or the validation cohort (n = 501). The application of the ALS specific cTnT cut‐off (B, D) reduced the number of biomarker‐negative patients and increased the proportion of patients with either isolated or combined biomarker elevations. [Color figure can be viewed at www.annalsofneurology.org]

In the context of myocardial assessment, a pathological threshold of 14ng/L well established to discern between elevated and non‐elevated serum cTnT levels.^11^ For the present study, we optimized the cut‐off value to best discriminate between ALS and non‐ALS cases. The Youden index derived from our ROC analysis allowed us to balance sensitivity and specificity of a threshold and optimize the diagnostic performance of the cTnT assay for ALS.^23^ The optimal threshold was 8.35ng/L (Youden index = 0.511), some 40% lower than the cardiac cut‐off (14ng/L). Using the conventional cardiac cut‐off, 92.2% of ALS patients were identified as biomarker‐positive. With the ALS‐adjusted cut‐off, this proportion increased to 96.3%. Applying this lower cut‐off improved the diagnostic sensitivity and led to the identification of 12 additional ALS patients (4.1%) with elevated cTnT levels (Fig 1A, B). To further validate this threshold, we combined the discovery cohort with the independent validation cohort (n = 794) and performed ROC analysis for cTnT as well. The optimal cut‐off identified in this combined dataset was again 8.35ng/L, yielding a Youden index of 0.5614, therefore, supporting the robustness and reproducibility of the proposed ALS‐specific threshold. Applying this threshold to the validation cohort, we classified 34 additional ALS patients (6.8%) as biomarker‐positive—a slightly higher proportion compared to the discovery cohort (see Fig 1C, D).

**FIGURE 2:**
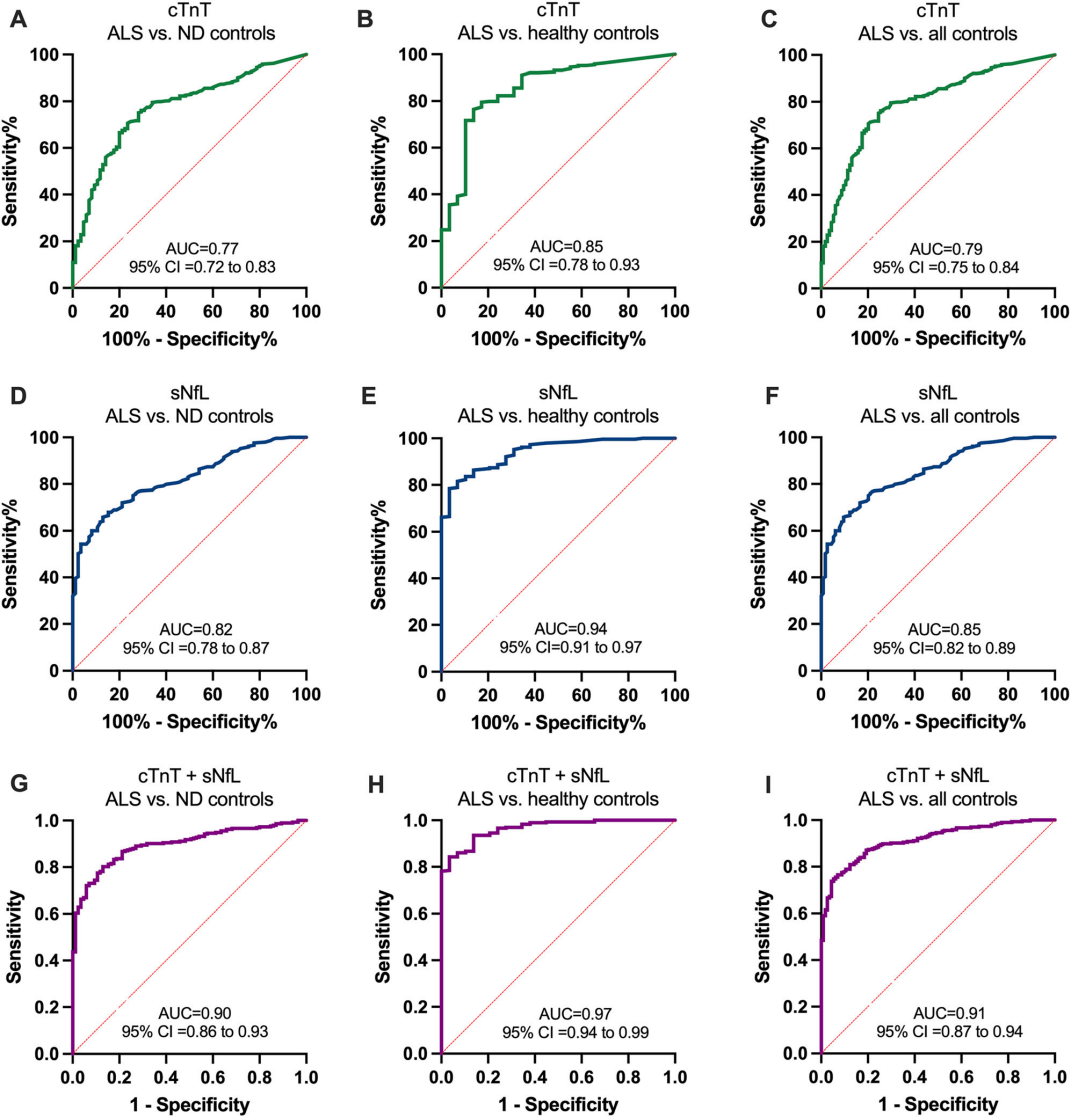
Receiver operating characteristic (ROC) curves of cardiac troponin T (cTnT), serum neurofilament light chain (sNfL), and their combination in the discovery cohort with a calculated area under the curve (AUC) and 95% confidence interval (95% CI). (A) ROC curve illustrates cTnT (green line) association with amyotrophic lateral sclerosis (ALS) diagnosis in comparison to neurodegenerative disease controls (ND controls). (B) ROC curve illustrates cTnT (green line) association with ALS diagnosis in comparison to healthy controls and was used to derive the Youden Index.  (C) ROC curve illustrates cTnT (green line) association with ALS diagnosis in comparison to all controls. (D) ROC curve illustrates sNfL (blue line) association with ALS diagnosis in comparison to ND controls. (E) ROC curve illustrates sNfL (blue line) association with ALS diagnosis in comparison healthy controls. (F) ROC curve illustrates sNfL (blue line) association with ALS diagnosis in comparison to all controls. (G) ROC curve illustrates cTnT + sNfL (purple line) association with ALS diagnosis in comparison to ND controls. (H) ROC curve illustrates cTnT + sNfL (purple line) association with ALS diagnosis in comparison to healthy controls. (I) ROC curve illustrates cTnT + sNfL (purple line) association with ALS diagnosis in comparison to all controls. All curves were calculated with *p* value of <0.0001. [Color figure can be viewed at www.annalsofneurology.org]

**FIGURE 3:**
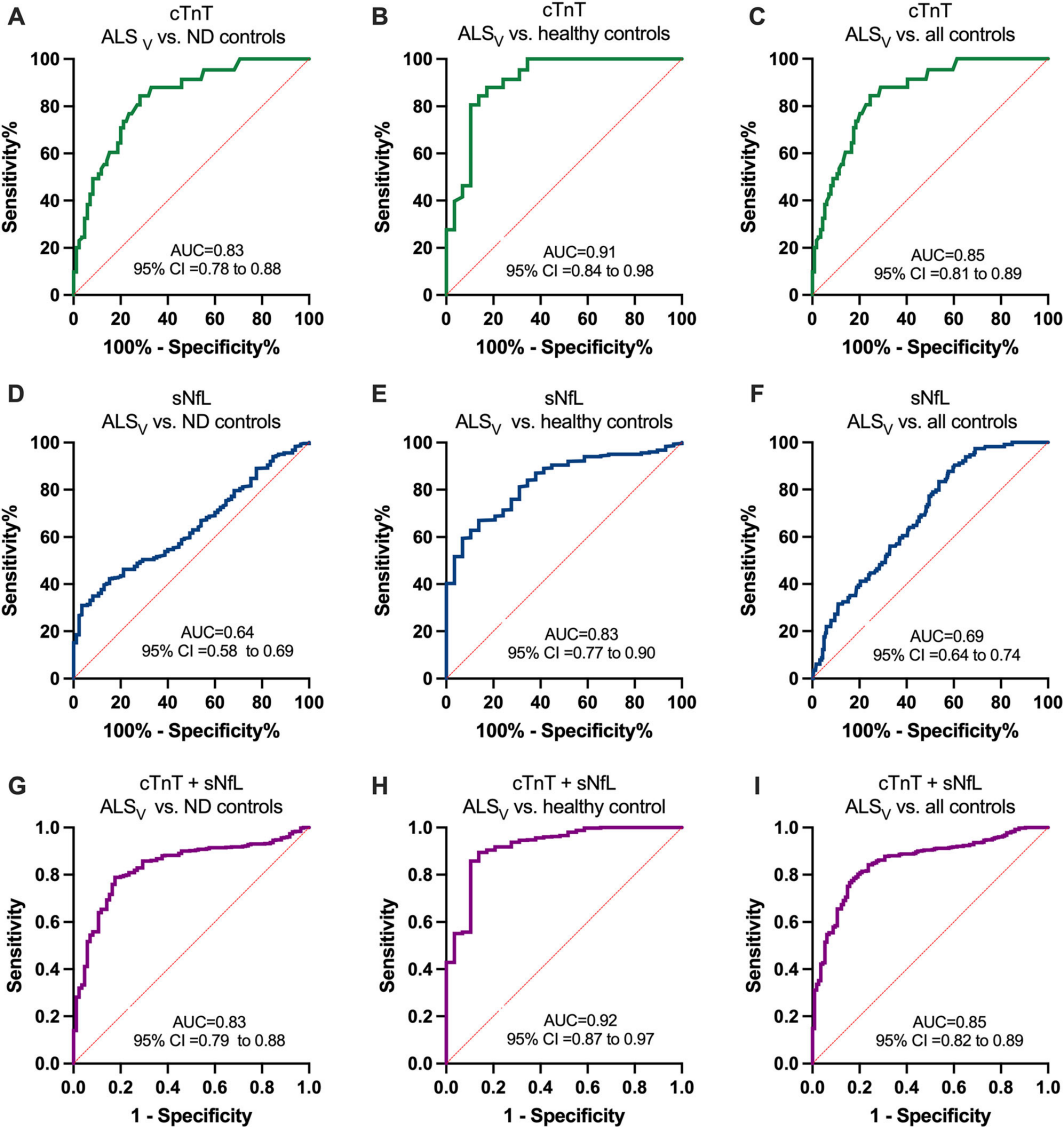
Receiver operating characteristic (ROC) curves of cardiac troponin T (cTnT), serum neurofilament light chain (sNfL), and their combination in the validation cohort with a calculated area under the curve (AUC) and 95% confidence interval (95% CI). (A) ROC curve illustrates cTnT (green line) association with amyotrophic lateral sclerosis (ALS) diagnosis in comparison to neurodegenerative disease controls (ND controls). (B) ROC curve illustrates cTnT (green line) association with ALS diagnosis in comparison to healthy controls. (C) ROC curve illustrates cTnT (green line) association with ALS diagnosis in comparison to all controls. (D) ROC curve illustrates sNfL (blue line) association with ALS diagnosis in comparison to ND controls (*p* = 0.05). (E) ROC curve illustrates sNfL (blue line) association with ALS diagnosis in comparison healthy controls. (F) ROC curve illustrates sNfL (blue line) association with ALS diagnosis in comparison to all controls. (G) ROC curve illustrates cTnT + sNfL (purple line) association with ALS diagnosis in comparison to ND controls (H) ROC curve illustrates cTnT + sNfL (purple line) association with ALS diagnosis in comparison to healthy controls. (I) ROC curve illustrates cTnT + sNfL (purple line) association with ALS diagnosis in comparison to all controls. All curves were calculated with *p* value of <0.0001, unless otherwise is stated. [Color figure can be viewed at www.annalsofneurology.org]

When comparing ALS patients of the discovery cohort with healthy controls, serum NfL achieved an AUC of 0.94 (95% CI: 0.91–0.97, *p* < 0.0001), whereas cTnT alone yielded an AUC of 0.85 (95% CI: 0.78–0.93, *p* < 0.0001). When combined, sNfL and cTnT produced an AUC of 0.97 (95% CI: 0.94–0.99, *p* < 0.0001), which was higher than the AUCs of the individual biomarkers. When comparing these ALS patients with neurodegenerative disease controls, serum NfL alone reached an AUC of 0.82 (95% CI: 0.78–0.87, *p* < 0.0001), and cTnT alone yielded an AUC of 0.77 (95% CI: 0.72–0.83, *p* < 0.0001). The combined use of sNfL and cTnT resulted in an AUC of 0.90 (95% CI: 0.86–0.93, *p* < 0.0001). When ALS patients were compared with all control subjects combined, the AUC for the combined biomarkers was 0.91 (95% CI: 0.87–0.94, *p* < 0.0001) (Fig 2). These results were further supported by data from the validation cohort, which showed a largely consistent diagnostic performance. When comparing ALS patients with healthy controls, sNfL achieved an AUC of 0.83 in the validation cohort, and cTnT reached and AUC of 0.91 (vs 0.85). The combination of both biomarkers showed an AUC of 0.92 (vs 0.97). In the comparison with neurodegenerative disease controls, sNfL achieved an AUC of 0.64 (vs 0.82), cTnT an AUC of 0.83 (vs 0.77) and the combination 0.83 (vs 0.90). When compared to all controls combined the AUCs in the validation cohort were 0.69 for sNfL (vs 0.85), 0.85for cTnT (vs 0.79), and 0.85 (vs 0.91) for combined biomarkers (Fig 3).

In addition, we analyzed the subgroup of ALS patients with normal levels of both biomarkers (biomarker‐negative; neither sNfL nor cTnT elevated) and compared them to ALS patients with elevated biomarker levels (biomarker‐positive; sNfL and/or cTnT elevated). Biomarker‐negative ALS patients were rare (n = 11, 3.8%) and exhibited a significantly longer median disease duration (73.0 months, IQR: 26.0–129.0) than biomarker‐positive patients (n = 282, 18.0 months, IQR: 9.00–31.0, *p* = 0.0003). The biomarker‐negative group had a significant slower median progression rate of 0.19 points per month (IQR: 0.06–0.36), compared to 0.70 points per month (IQR: 0.36–1.25) in biomarker‐positive patients (*p* < 0.0001) (Table 2). We also conducted a subgroup analysis within the validation cohort comparing biomarker‐positive and biomarker‐negative ALS patients. Although disease duration did not show a significant difference between these subgroups, biomarker‐negative patients (n = 46, 9.2%) exhibited a significantly lower progression rate and higher ALSFRS‐R score (Table 2).

**Table 2:** Comparison between Biomarker‐Positive and Biomarker‐Negative ALS patients in the Discovery and the Validation Cohort

	Discovery cohort			Validation cohort		
	Biomarker‐positive	Biomarker‐negative	*p*	Biomarker‐positive	Biomarker‐negative	*p*
n	282	11	NA	455	46	NA
Sex, F, n (%)	120 (42.6)	6 (54.5)	NA	180 (39.6)	23 (50)	NA
Age, yr	67 (59–75)	65.0 (61.0–76.0)	0.9180	64 (56.5–72) n = 449	54.0 (42.5–62.0) n = 41	<0.0001
BMI (kg/m^2^)	24.1 (22.1–26.8) n = 224	25.12 (21.28–26.68) n = 10	0.8223	NA	NA	NA
ALSFRS‐R	34.0 (28.0–39.0) n = 235	41.0 (35.3–43.5) n = 8	0.0396	34.0 (28.0–40.0) n = 448	44.0 (39.0–48.0)	<0.0001
Disease duration, months	18.0 (9.00–31.0) n = 273	73.0 (26.0–129.0)	<0.0003	7.00 (2.00–25.0) n = 448	6.00 (2.00–34.5) n = 41	0.7823
Progression rate, points per month	0.70 (0.36–1.25) n = 232	0.19 (0.06–0.36) n = 8	<0.0001	1.36 (0.5–4.33) n = 419	0.27 (0.06–1.075) n = 36	<0.0001
cTnT median value (ng/L)	19.2 (9.58–34.9)	4.9 (3.00–6.10)	<0.0001	22.0 (13.0–39.0)	6.00 (5.00–7.00)	<0.0001
sNfL median value (pg/mL)	97.5 (58.0–152.3)	22.0 (14.0–40.0)	<0.0001	58.7 (33.3–99.4)	11.36 (5.9–23.2)	<0.0001

All values are presented as median (interquartile range). *p* Values were calculated using the Mann–Whitney *U* test. Biomarker‐positive patients had elevated sNfL and/or cTnT levels using the ALS‐specific cTnT cut‐off (8.35ng/L); biomarker‐negative patients had normal levels of both biomarkers.

ALSFRS‐R = Revised Amyotrophic Lateral Sclerosis Functional Rating Scale; BMI = body mass index; cTnT = cardiac troponin T; F = female; NA = not applicable; sNfL = serum neurofilament light chain; yr = years.

## Discussion

ALS patients, as shown in previous studies,^15^ exhibit elevated levels of both sNfL and cTnT, a finding confirmed in our study. Individually, each biomarker demonstrated good diagnostic accuracy, but their combination led to a substantial increase in discriminatory performance. This improvement was particularly pronounced when distinguishing ALS patients from the neurodegenerative controls. Of note, this control group was composed of both non‐ALS motoneuron and non‐motoneuron neurodegenerative disease cases, namely HD, providing confidence for the generalizability in clinical practice.

Consistent with previous literature, we observed excellent discriminatory power of sNfL for differentiating ALS from healthy controls.^4^, ^24^ However the performance of sNfL alone was reduced, when comparing ALS with neurodegenerative disease controls. This observation highlights a known limitation and aligns with earlier studies showing variable discriminative performance depending on the control cohort.^5^ On this basis, our validation cohort provided additional support for the observed limitations of sNfL and the complementary value of cTnT. Despite demographic and clinical differences—such as younger age, shorter disease duration, and higher progression rate—the main findings were reproduced. Although sNfL showed good discrimination between ALS patients and healthy controls, its performance in differentiating ALS from neurodegenerative disease controls was notably lower in the validation cohort (AUC = 0.64) than in the discovery cohort (AUC = 0.82). This difference further illustrates the limitations of using sNfL alone for differential diagnosis in this context.

The addition of cTnT—conventionally a marker of myocardial injury, but increasingly recognized as a sign of skeletal muscle pathology—substantially enhanced diagnostic performance. These findings are in line with results reported by Castro‐Gomez et al,^15^ and by Kläppe et al^16^ who observed cTnT elevations in ALS, although the latter did not report a significant gain from combining biomarkers, potentially because of differences in control group composition and sample size. The comparative study by Vidovic et al^3^ also highlights the growing importance of muscle biomarkers as a complement to neurofilaments in the biomarker profiling of ALS. Importantly, based on ROC analysis and the Youden index, we identified an optimized diagnostic cut‐off for cTnT at 8.35ng/L—well below the conventional cardiac reference limit of 14.0ng/L.^11^ This lower threshold increases sensitivity and may allow for earlier identification of ALS‐related skeletal muscle involvement. Our data shows that using this new cut‐off helps identify a subset of ALS patients who fall below the conventional (“cardiac”) threshold. In our discovery cohort, 3.5% of ALS patients were newly identified as biomarker‐positive when applying the adjusted cut‐off, supporting its potential diagnostic relevance. This effect was even more pronounced in the validation cohort, where 6.8% of patients were newly classified as biomarker‐positive. Further validation in independent cohorts is warranted.

Another key finding of our study was the identification of a small subgroup of biomarker‐negative ALS patients (with neither elevated sNfL nor cTnT). These patients, although rare (3.8% in the discovery cohort and 9.2% in the validation cohort), showed significantly slower disease progression, longer disease duration, and a higher ALSFRS‐R compared to biomarker‐positive individuals. This may correspond to a clinically distinct, more slowly progressive ALS subgroup. Our results are in line with earlier studies linking lower sNfL levels to reduced disease aggressiveness and may also support recent findings associating elevated cTnT with respiratory decline and worse prognosis.^6^, ^25^ In conclusion, our findings support the use of sNfL and cTnT as complementary biomarkers in ALS. Their combined assessment enhances diagnostic accuracy and may enable earlier detection and characterization of distinct subgroups with prognostic implications. A practical advantage of the repurposing of a well‐established biomarker that is in wide clinical use (albeit in a different context) is that questions like scalability, accessibility, and reproducibility of the assay across different settings is firmly settled.^26^ The newly proposed cTnT cut‐off may represent a valuable diagnostic tool, although further prospective and longitudinal studies are needed to confirm its clinical utility and prognostic significance. Further research should examine the diagnostic stability of this cut‐off across diverse cohorts and explore the clinical features of biomarker‐negative subtypes, as well as the temporal dynamics of biomarker levels in relation to disease progression and survival.

## Author Contributions

P.W., and S.B. conceived of and designed the study. P.L., R.F., T.G., H.N., T.M., S.B., and T.M. contributed to acquisition and analysis of the data. P.L., S.B., and P.W. drafted the text and prepared the figures. [Correction added on 09 February 2026, after first online publication: Author contribution text has been revised in this version.]

## Potential Conflicts of Interest

Nothing to report.

## Data Availability

All data will be made available on reasonable request to the corresponding author, P.W.
